# Supplementary material for: Dietary Factors and Risks of Cardiovascular Diseases: An Umbrella Review
Source: Nutrients. 2020 Apr 15;12(4):1088. doi: 10.3390/nu12041088 (PMC7231110; doi:10.3390/nu12041088)
Supplement: Supplementary file 1 [file nutrients-12-01088-s001.pdf]

Appendix S1: Search terms and search strategies from Scopus and Medline databases.

Scopus

((TITLE-ABS-KEY ("prevent*")) OR (TITLE-ABS-KEY ("delay*")) OR (TITLE-ABS-KEY ("reduc*"))) AND ((TITLE-ABS-KEY ("systematic review")) OR (TITLE-ABS-KEY ("meta analysis"))) AND ((TITLE-ABS-KEY ("lifestyle change" OR "lifestyle modification" OR "lifestyle adaptation" OR "lifestyle intervention" OR "lifestyle therapy" OR "lifestyle treatment")) OR (TITLE-ABS-KEY ("behavior change" OR "behavior modification" OR "behavior adaptation" OR "behavior intervention" OR "behavior therapy" OR "behavior treatment" OR "non-pharmacological*" OR "nonpharmacological*")) OR (((TITLE-ABS-KEY ("low salt")) OR (TITLE-ABS-KEY ("weight reduction")) OR ((TITLE-ABS-KEY ("carbohydrate-restricted diet")) OR (TITLE-ABS-KEY ("Mediterranean diet")) OR (TITLE-ABS-KEY ("fat-restricted diet")) OR (TITLE-ABS-KEY ("reducing diet")) OR (TITLE-ABS-KEY ("diet therapy")) OR (TITLE-ABS-KEY ("diet* control")) OR (TITLE-ABS-KEY ("dietary control"))) OR ((TITLE-ABS-KEY ("low glycemic index")) OR (TITLE-ABS-KEY ("low carbohydrate")) OR (TITLE-ABS-KEY ("dietary approach to stop hypertension")) OR (TITLE-ABS-KEY ("healthy eat*")) OR (TITLE-ABS-KEY ("clean food")) OR (TITLE-ABS-KEY ("salt restriction")))) OR ((TITLE-ABS-KEY ("strength training")) OR (TITLE-ABS-KEY ("weight training")) OR ((TITLE-ABS-KEY ("physical activity")) OR (TITLE-ABS-KEY (sport)) OR (TITLE-ABS-KEY (exercise)) OR (TITLE-ABS-KEY (workout)) OR (TITLE-ABS-KEY ("work out")) OR (TITLE-ABS-KEY ("resistance training")))) OR ((TITLE-ABS-KEY ("smoking cessation")) OR (TITLE-ABS-KEY ("smoking quit*")) OR (TITLE-ABS-KEY ("smoking reduc*")) OR (TITLE-ABS-KEY ("tobacco cessation")) OR (TITLE-ABS-KEY ("tobacco quit*")) OR (TITLE-ABS-KEY ("tobacco reduc*"))) OR ((TITLE-ABS-KEY ("alcohol cessation")) OR (TITLE-ABS-KEY ("alcohol reduc*"))) OR ((TITLE-ABS-KEY (sleep)) OR (TITLE-ABS-KEY ("stress reduc*"))))) AND ((TITLE-ABS-KEY ("cardiovascular disease" OR "coronary artery disease" OR "acute coronary syndrome" OR "myocardial infarction")) OR (TITLE-ABS-KEY ("ischemic heart disease" OR "angina pectoris" OR "unstable angina" OR stroke OR "cerebrovascular disease" OR "cardiovascular death" OR "cardiovascular mortality")))

Medline

Search ((((((((((("Cardiovascular Diseases"[Mesh]) OR "cardiovascular disease") OR "coronary artery disease") OR "acute coronary syndrome") OR "myocardial infarction") OR "ischemic heart disease") OR stroke) OR "cerebrovascular disease") OR "cardiovascular death") OR "cardiovascular mortality")) AND (((((((((((((((("Life Style"[Mesh]) OR “lifestyle change”) OR “lifestyle modification”) OR “lifestyle adaptation”) OR “lifestyle intervention”) OR “lifestyle therapy”) OR “lifestyle treatment”) OR “behavior change”) OR “behavior modification”) OR “behavior adaptation”) OR “behavior intervention”) OR “behavior therapy”) OR “behavior treatment”)) OR (((((((((((((("non-pharmacological*") OR "nonpharmacological*") OR (("non-pharmacological*") OR "nonpharmacological*")) OR "Diet, Carbohydrate-Restricted"[Mesh]) OR "Diet, Mediterranean"[Mesh]) OR "Diet, Fat-Restricted"[Mesh]) OR "Diet, Reducing"[Mesh]) OR "Diet Therapy"[Mesh]) OR "diet* control") OR “dietary control”) OR "low glycemic index") OR "low carbohydrate") OR "dietary approach to stop hypertension") OR "healthy eat*") OR "clean food")) OR ((((((("physical activity") OR exercise) OR sport) OR workout) OR "work out") OR "resistance training") OR "strength training") OR "weight training")) OR ((((((("Smoking Cessation"[Mesh]) OR “smoking cessation”) OR "smoking quit*") OR "smoking reduc*") OR "Tobacco Use Cessation"[Mesh]) OR "tobacco cessation") OR "tobacco quit*") OR "tobacco reduc*")) OR ((("alcohol cessation") OR "alcohol reduc*") OR (("alcohol cessation") OR "alcohol reduc*"))) OR (((sleep) OR (("Stress, Psychological/prevention and control"[Mesh]))) OR “stress reduc*”))) AND (((prevent*) OR delay*) OR reduc*) Filters: Systematic Reviews

Table S1. Characteristics of included systematic reviews and meta-analyses.

Author	Year	Population	Intervention/ factor	Outcome	Type of included studies	Age	% Male	Total *n*	Setting
					Food patterns				
Onvani[1]	2016	General population	HEI and AHEI	All-causes and, CVD mortality	Cohort study: 12<br>Cross-sectional: 1	18–82	0–100	636–424,663	US: 11<br>UK: 1<br>Asian: 1
Semlitsch[2]	2016	HT patients with obesity	Diet intervention	All-causes mortality, CVD	Parallel RCT: 4<br>Cluster RCT: 3<br>Factorial RCT: 1	47–66	27–64	30–587	US: 4<br>UK: 1<br>European: 2<br>Unknown: 1
Guo[3]	2017	General population	CVH score	All-causes and CVD mortality, CVD	Cohort: 13	43–69	36.3–100	2981–95,429	US: 7<br>UK: 1<br>Asian:5
Schwingshackl[4]	2018	General population	HEI, AHEI, and DASH score	All-causes and CVD mortality, CVD	Cohort: 68	18–104	0–100	298–460,770	US: 26<br>UK: 1<br>European: 3<br>Asian: 2<br>Australia: 1<br>Multi-centre: 1
Shivappa[5]	2018	General subject	DII score	CVD	Cohort: 11<br>Cross-sectional: 1<br>Case-control: 1<br>Nested case-control: 1	NR	0–100	171–3725	US: 3<br>UK: 1<br>European: 7<br>Australia: 3
Sofi[6]	2008	General population	Mediterranean diet	All-causes and CVD mortality	Cohort: 12	20–90	0–100	161–214,284	US: 4<br>European: 7<br>Australia: 1
Sofi[7]	2010	General population	Mediterranean diet	All-causes and CVD mortality, CVD	Cohort: 7	20–95	0–100	1199–485,044	US: 2<br>European: 5
Psaltopoulou[8]	2013	General population	Mediterranean diet	Stroke	Cohort: 9<br>Case-control: 3<br>Cross-sectional: 10	37.2–80.3	0–72	575–74,886	US: 9<br>UK: 1<br>European: 10<br>Australia: 2
Martínez-González[9]	2014	General population	Mediterranean diet	CVD	RCT: 2<br>Cohort: 12	NR	NR	NR	NR
Kontogianni[10]	2014	General population	DASH, Mediterranean diet	Stroke	NR	NR	NR	NR	NR

Author	Year	Population	Intervention/ factor	Outcome	Type of included studies	Age	% Male	Total *n*	Setting
Liyanage[11]	2016	High risk of CVD (i.e. DM)	Mediterranean diet	All-causes and CVD mortality, CVD	Parallel RCT: 6	37–41	0–100	48–7447	US: 1<br>UK: 1<br>European: 2<br>Asian: 2
Grosso[12]	2017	General population	Mediterranean diet	CVD mortality, CVD	Cohort: 20<br>Parallel RCT: 4<br>Case-control: 4	48–67.3	0–100	605–380,296	US: 5<br>European: 17<br>Asian: 1<br>Australia: 1
Rosato[13]	2019	General population	Mediterranean diet	CHD/MI, Stroke. CVD	Cohort: 21<br>Case-control: 5	20–92	0–100	46–347,000	US:7<br>UK: 1<br>European: 19<br>Asian:1<br>Australia:1
Chen[14]	2019	General population	Mediterranean diet	Stroke	Cohort: 20	42–72	NR	NR	US: 6<br>European: 11<br>UK: 1<br>Asian: 2
				Food patterns: DASH diet					
Abargouei[15]	2013	General population	DASH diet	CVD mortality, CVD	Cohort: 6	35–79	0–100	20933–88,517	US: 3<br>European: 3
Kwok[16]	2014	General population	Vegetarian diet	CVD mortality, CVD	Cohort: 8	25–100	24–100	1904–73,308	US: 2<br>UK: 3<br>European: 2<br>Asian: 1
Feng[17]	2018	General population	DASH diet	Stroke	Prospective Cohort: 12	20–83	0–100	1867–172,043	US:4<br>UK:2<br>European:4<br>Asian:2
Yang[18]	2019	General population	DASH diet	CHD	Cohort: 7	20–79	0–100	1867–153,802	US: 4<br>UK: 2<br>European: 1
				Food groups					
Law[19]	1998	General population	Fruit and vegetable intakes	CHD	Cohort: 11	25–75	0–100	343–87,245	US: 5<br>UK: 3<br>European: 2
Hu[20]	2014	General population	Fruit and vegetable intakes	Stroke	Cohort: 20	40.7–72.4	NR	552–174,888	US: 6<br>European: 8<br>Asian: 6
Whelton[21]	2004	General population	Fish intake	CHD	Cohort: 14<br>Case-control: 5	22–87	0–100	272–84,688	US: 8<br>European: 9

Author	Year	Population	Intervention/ factor	Outcome	Type of included studies	Age	% Male	Total *n*	Setting
									Asian: 2
Crippa[22]	2014	General population	Coffee intake	All-causes and CVD mortality	Cohort: 21	NR	0–100	1440–45,4775	US: 8<br>UK: 2<br>European: 7<br>Asian: 4
Kwok[23]	2015	General population	Chocolate intake	CVD	Cohort: 7<br>Parallel RCT: 1<br>Cross-sectional: 1	49–75	0–100	470–37,103	US: 2<br>UK: 1<br>European: 5<br>Australia: 1
Yuan[24]	2017	General population	Chocolate intake	CVD	Cohort: 14	35–84	0–100	590–92,678	US: 5<br>UK: 1<br>European: 5<br>Asian: 2<br>Australia: 1
Pang[25]	2015	General population	Green tea consumption	All-causes and CVD mortality, CVD	Cohort: 8<br>Parallel RCT: 1	40–89	0–72	51–100,938	NR
Shao[26]	2016	General population	Nut intake	Stroke	Cohort: 11	30–86.7	0–100	2893–120,852	US: 6<br>European: 2<br>Asian: 2<br>Australia: 1
Weng[27]	2016	General population	Nut intake	CVD	Cohort: 13<br>Parallel RCT: 1	24–80	NR	6309–86,016	US: 12<br>European: 2
Wei[28]	2016	General population	Whole grain intake	All-causes and CVD mortality	Cohort: 11	42–72	0–100	535–367,442	US: 8<br>European: 3
Marventano[29]	2016	General population	Legume intake	CVD	Cohort: 14	20–86	0–100	3932–84,136	US: 6<br>European: 5<br>Asian: 3
Food nutrients									
Hooper[30]	2000	General population	Low or modified fat diet	All-causes and CVD mortality	RCT: 27	25–88	0–100	21–9032	NR
Studer[31]	2005	General population	Diet intervention, n-3 PUFA	All-causes and CVD mortality	RCT: 31	NR	NR	NR	NR
Skeaff[32]	2009	General population	Fat intake	CVD mortality, CHD	NR	NR	NR	NR	NR
Mozaffarian[33]	2010	General population	n-6 PUFA	CVD mortality	RCT: 8	NR	0–100	55–9057	NR
Ramsden[34]	2010	General population	PUFA, n-6 PUFA	All-causes and CVD mortality	RCT: 7	NR	NR	55–9057	NR

Author	Year	Population	Intervention/ factor	Outcome	Type of included studies	Age	% Male	Total *n*	Setting
Lista[35]	2012	General population	Marine omega-3 PUFA	All-causes and CVD mortality, CVD	RCT: 21	NR	NR	NR	NR
Abdelhamid[36]	2019	General population	omega-3 PUFA	All-causes and CVD mortality, CVD, CHD, stroke	RCT: 79	NR	NR	11–18,645	NR
Martínez-González[37]	2014	General population	Olive oil	CHD, stroke	Case-control: 3<br>RCT: 1<br>Cohort: 5	49–78	NR	342–40,142	European: 9
Schwingshackl[38]	2014	General population	MUFA, olive oil, oleic acid	All-causes and CVD mortality, CVD, CHD, stroke	Cohort: 42	20–87	0–100	161–161,808	US: 13<br>European: 20<br>UK: 1<br>Israel: 1<br>Asian: 4<br>Australia: 1
Farvid[39]	2015	General population	Dietary linoleic consumption	CHD	Cohort: 13	35–61	0–100	1643–84,564	US: 6<br>European: 6<br>Asian: 1
Harcombe[40]	2016	General population	Low or modified fat diet	All-causes and CVD mortality	RCT: 10	NR	0–100	52–48,835	NR
Harcombe[41]	2017	General population	Dietary fat intake	CVD mortality	Cohort: 7	30–79	39–100	2676–43,757	NR
Muto[42]	2018	General population	Saturated Fatty acid	stroke	Cohort: 11	34–89	0–100	832– 87,025	US: 4<br>European: 2<br>Asian: 5
Hollman[43]	2010	General population	Flavonoid intake	Stroke	Cohort: 6	39.3–61.5	0–100	NR	US: 2<br>European: 4
Kim[44]	2017	General population	Flavonoid intake	All-causes and CVD mortality, CHD	Cohort: 15	30–84	0–100	805–66,360	US: 4<br>UK: 1<br>European: 8<br>Asian: 1<br>Australia: 1
Kimble[45]	2019	General population	Flavonoid intake (Anthocyanins)	CVD, CVD mortality, CHD, stroke	Cohort: 19	25–75	0–100	NR	US: 7<br>European: 11<br>Australia: 1
D'Elia[46]	2011	General population	Potassium intake	Stroke, CVD, CHD	Cohort: 11	25–79	0–100	859–58,730	US: 6<br>European: 3<br>Asian: 2
Chen[47]	2013	General population	Dietary fibre intake	Stroke	Cohort: 6	30–79	0–100	20674–86,387	US: 2<br>European: 2<br>Asian: 2

Author	Year	Population	Intervention/ factor	Outcome	Type of included studies	Age	% Male	Total *n*	Setting
Kim[48]	2016	General population	Dietary fibre intake	CVD mortality, CHD	Cohort: 15	20–85	0–100	859–388,122	US: 5<br>European: 7<br>Asian: 1<br>Australia: 2
Li[49]	2017	General population	Dietary fibre intake	Ischemic stroke	Cohort: 8	30–83	0–100	1772–96,677	US: 3<br>European: 3<br>Asian: 2
Reynolds[50]	2019	General population	Whole grain, Dietary fibre intake	All-cause mortality CVD mortality CHD, stroke	Cohort:185<br>RCT: 58	18–73	0–100	7–40,067	NR
				Food nutrients: Salt diet					
Aburto[51]	2013	General population and HT patients	Low salt diet	All-causes mortality, CVD, CHD	Parallel RCT: 42<br>Cohort: 14	NR	NR	16–2382	NR
Adler[52]	2014	General population and HT patients	Low salt diet	All-causes and CVD mortality, CVD	Parallel RCT: 6<br>Cluster RCT: 2	38.5–83.3	22.1–100	NR	Asian: 3<br>Australia: 1
Jayedi[53]	2019	General population	Low salt diet	Stroke	Cohort: 14<br>Case-cohort: 2	48–69	NR	464–77,500	US: 6<br>European: 4<br>Asian: 5<br>International: 1
				Food nutrients: Dietary calcium					
Wang[54]	2014	General population	Dietary calcium intake	CVD mortality	Cohort:11	4–74	0–100	2605–388,229	US: 3<br>European: 4<br>Asian: 4<br>Canada: 1

AHEI: alternate healthy eating index; CVD: cardiovascular diseases; CHD: coronary heart disease; CVH: cardiovascular health; DII: dietary inflammatory index; DM: diabetes mellitus; DASH: Dietary Approaches to Stop Hypertension; HT: hypertension; HEI: healthy eating index; NR: not reported; MUFA: monounsaturated fatty acid; PUFA: polyunsaturated fatty acid; RCT: randomized-controlled trial; US: United States; UK: United Kingdom.

Table S2. Details of diet intervention and dietary factors.

	Year	Intervention/Exposure	Mode of deliver/type of questionnaire	Setting	Behavioral intervention (BI)	Support device	Duration
			Food Patterns: Healthy diet				
Onvani[1]	2016	Healthy diet	HEI, AHEI score	NA	NA	NA	6.2–22 years
Semlitsch[2]	2016	Low CBH, low fat, low calorie diet; increased fish, vegetables, complex CBH, and fiber consumption	Supervised and education	Clinic-based	No	No	0.5–3 years
Guo[3]	2017	Healthy diet	CVH score	NA	NA	NA	4–18.7 years
Schwingshackl[4]	2018	Healthy diet	HEI, AHEI, DASH score	NA	NA	NA	3–24 years
Shivappa[5]	2018	Healthy diet	Dietary inflammatory index	NA	NA	NA	4.3–26 years
			Food Patterns: Mediterranean diet				
Sofi[6]	2008	Mediterranean diet	Mediterranean diet adherence score (0–9)	NA	NA	NA	3.7–18 years
Sofi[7]	2010	Mediterranean diet	Mediterranean diet adherence score (0–9)	NA	NA	NA	4.5–20 years
Psaltopoulou[8]	2013	Mediterranean diet	Mediterranean diet adherence score (0–9)	NA	NA	NA	2.2–20 years
Martínez-González[9]	2014	Mediterranean diet	The Mediterranean Diet Score	NA	NA	NA	NR
Kontogianni[10]	2014	Mediterranean and DASH diet	Validated FFQ	NA	NA	NA	NR
Liyanage[11]	2016	Mediterranean diet	Education and supplement	Clinic based	No	No	6 months – 9 years
Grosso[12]	2017	Mediterranean diet assessed by mMed, tMed, aMed, rMed, MedDiet, MeDi, IMed score	Supervised and educational	Clinic and home based	No	No	RCTs: 2–5 years Observational studies: 2–20 years
Chen[14]	2019	Mediterranean diet	FFQ, 7-d food record, 14-point Med-Diet adherence screener	NA	NA	NA	3–21 years
Rosato[13]	2019	Mediterranean diet	Mediterranean diet score	NA	NA	NA	6–20 year
			Food Patterns: DASH diet				
Abargouei[15]	2013	DASH diet	quintiles of eight DASH dietary pattern components	NA	NA	NA	7–24 years
Kontogianni[10]	2014	Mediterranean and DASH diet	validated FFQ	NA	NA	NA	NR
Feng[17]	2018	DASH diet	FFQ	NA	NA	NA	5.7–24 years
Yang[18]	2019	DASH diet	FFQ	NA	NA	NA	2.8–24 years
			Food Patterns: Vegetarians diet				
Kwok[16]	2014	Vegetarian diet	NR	NA	NA	NA	6–23 years
			Food groups: Fruit and vegetable				
Law[19]	1998	Fruit and vegetable	FFQ, 7 day weighted inventory, 4 week and 1 year diet history	NA	NA	NA	4–24 years
Hu[20]	2014	Fruit and vegetable	FFQ, 24 hr food recall	NA	NA	NA	3–37 years
			Food groups: Fish intake				

	Year	Intervention/Exposure	Mode of deliver/type of questionnaire	Setting	Behavioral intervention (BI)	Support device	Duration
Whelton[21]	2004	Fish intake	Analyzed based on the number of servings per week	NA	NA	NA	4–30 years
			Food groups: Coffee				
Crippa[22]	2014	Coffee intake	NR	NA	NA	NA	7.1–24 years
			Food groups: Chocolate				
Kwok[23]	2015	Chocolate intake	FFQ	NA	NA	NA	8–16 years
Yuan[24]	2017	Chocolate intake	FFQ and self-report	NA	NA	NA	4.7–16 years
			Food groups: Green tea				
Jun Pang[25]	2015	Green tea intake	NR	NA	NA	NA	9 weeks – 13 years
			Food groups: Nut				
Shao[26]	2016	Nut intake	FFQ	NA	NA	NA	4.4–30 years
Weng[27]	2016	Nut intake	NR	NA	NA	NA	4.8–26 years
			Food groups: Whole grain				
Wei[28]	2016	Whole grain	FFQ	NA	NA	NA	5.5–26 years
Reynolds[50]	2019	Whole grain, dietary fibre	RCT: meal supplement<br>Cohort: FFQ, 24-hr recall, 7 day dietary record	NA	NA	NA	6 weeks – 24 years
			Food groups: Legume				
Marventano[29]	2016	Legume consumption	NR	NA	NA	NA	4.9–26 years
			Food nutrients: Fat				
Hooper[30]	2000	Reduce fat intake 15%-30% of TE, replace SFA by linoleic acid, reduce cholesterol intake	Education, meal supplement	Clinic-based	NR	NR	0.43–9.3 years
Studer[31]	2005	n-3 PUFA<br>Dietary intervention	NR	NR	NR	NR	n–3 PUFA 1.2–1.9 years<br>diet 2.5–4.2 years
Skeaff[32]	2009	Fat intake	FFQ, 24 hr recall, weighed diet records, cross-check dietary history, dietary history, 4 day estimated food record, 7 day weighed diet record	NA	NA	NA	NR
Mozaffarian[33]	2010	Increased n-6 PUFA	Education, meal supplement	Clinic-based	No	No	1–8 years
Ramsden[34]	2010	Increased mixed n-3/n-6 PUFA, only n-6 PUFA	NR	NR	NR	NR	2–4 years
Lista[35]	2012	Marine omega-3 FA	NR	Clinic-based	NR	NR	1–9 years
Martínez-González[37]	2014	Olive oil	FFQ	NA	NA	NA	4.8–10.4 years
Schwingshackl[38]	2014	MUFA, MUFA: SFA, olive oil, Oleic acid	NR	NR	NR	NR	3.7–30 years
Farvid[39]	2015	Dietary linoleic acid	FFQ, 24 hr food recall, 7-day weight food record	NA	NA	NA	5.3–30 years

	Year	Intervention/Exposure	Mode of deliver/type of questionnaire	Setting	Behavioral intervention (BI)	Support device	Duration
Harcombe[40]	2016	64 g Corn oil, 58 g olive oil, 40 g fat/day, 85 g soya-bean oil, 20%–40% calories from fat, 8%–10% calories from SFA	NR	Clinic-based	NR	NR	Mean 4.7 years
Harcombe[41]	2017	Dietary fat	NR	NA	NA	NA	6–20 years
Muto[42]	2018	Saturated fat intake	FFQ and 24-hr food recall	NA	NA	NA	7.6–20 years
Abdelhamid[36]	2019	Omega-3 PUFA	Diet education, meal supplement, oil supplement	Clinic-based	NR	NR	1 year
			Food nutrients: Flavonoid				
Hollman[43]	2010	Flavonoid intake	Dietary history, FFQ, 4 day food record	NA	NA	NA	6.1–28 years
Kim[44]	2017	Flavonoid intake	FFQ, Interviews, 4 day food records	NA	NA	NA	4.8–28 years
Kimble[45]	2019	Flavonoid intake (Anothocyanins)	FFQ, Dietary history interview, 4 day food diary	NA	NA	NA	5–41 years
			Food nutrients: Potassium				
D'Elia[46]	2011	Potassium intake	FFQ, 24 h dietary recall, Overnight urine K, 24 h urine collection	NA	NA	NA	5–19 years
			Food nutrients: Dietary fiber				
Chen[47]	2013	Dietary fiber intake	NR	NA	NA	NA	8–18 years
Kim[48]	2016	Dietary fiber intake	FFQ, 24-hour dietary recall, cross-check dietary history method, 7-day food diary	NA	NA	NA	5.9–40 years
Li[49]	2017	Dietary fiber intake	FFQ	NA	NA	NA	8.6–18 years
			Food nutrients: Low salt				
Aburto[51]	2013	Low salt diet	Education, meal supplement	Clinic-based	NR	NR	1 month – 3 years
Adler[52]	2014	Low salt diet	Education, meal supplement	Clinic-based	Yes	No	1.5–3 years
Jayedi[53]	2019	Low salt diet	FFQ, 24-hour dietary recall, 24 h urinary sodium excretion, 3 day weighting dietary record	NA	NA	NA	4.9–28 years
			Food nutrients: Dietary calcium				
Wang[54]	2014	Dietary or supplement calcium	NR	NA	NA	NA	5.5–28 years

AHEI: alternate healthy eating index; CVD: cardiovascular diseases; CHD: coronary heart disease; CVH: cardiovascular health; DII: dietary inflammatory index; DASH: Dietary Approaches to Stop Hypertension; HT: hypertension; HEI: healthy eating index; NA: not applicable; NR: not reported; MUFA: monounsaturated fatty acid; PUFA: polyunsaturated fatty acid; RCT: randomized-controlled trial; SFA: saturated fatty acid.

Table S3: Pooled risk ratios of dietary factors according to outcomes.

	Year	Types of study	Population	No. of primary studies	Total n	Pooled RR (95% CI)	Heterogeneity, I^2	Publication bias	Risk of bias
				Healthy diet (all-cause mortality)					
Studer[31]	2005	Diet intervention (RCT)	General population	17	115310	0.97 (0.92–1.04)	23	NR	Critically low
Onvani[1]	2016	HEI and AHEI (highest vs lowest)	General population	12	NR	0.77 (0.76–0.78)	NR	No	Critically low
Guo[3]	2017	CVH score (highest vs lowest)	General population	6	NR	0.54 (0.41–0.69)	65.1	No	Critically low
Schwingshackl[4]	2018	HEI and AHEI (highest vs lowest)	General population	13	NR	0.78 (0.77–0.80)	59	No	Critically low
				Healthy diet (CVD mortality)					
Studer[31]	2005	Diet intervention (RCT)	General population	18	115310	0.91 (0.82–1.02)	27	NR	Critically low
S. Onvani[1]	2016	HEI and AHEI (highest vs lowest)	General population	9	NR	0.77 (0.74–0.80)	NR	No	Critically low
Leilei Guo[3]	2017	CVH score (highest vs lowest)	General population	6	NR	0.30 (0.18–0.51)	66.3	No	Critically low
Nitin Shivappa[5]	2018	DII (highest vs lowest)	General population	6	NR	1.37 (1.11–1.70)	77	NR	Critically low
				Healthy diet (CVD)					
Semlitsch[2]	2016	Diet intervention (RCT)	Obesity/overweight with HT patients	1	585	0.70 (0.57–0.87)	NR	NR	Low
Schwingshackl[4]	2018	HEI and AHEI (highest vs lowest)	General population	28	NR	0.78 (0.76–0.80)	49	P 0.16	Critically low
Shivappa[5]	2018	DII (highest vs lowest)	General population	6	NR	1.35 (1.11–1.63)	36	NR	Critically low
				Mediterranean diet (All-cause mortality)					
Sofi[6]	2008	Cohort (per 2 points increased in score)	General population	8	NR	0.91 (0.89–0.94)	48.8	NR	Critically low
Sofi[7]	2010	Cohort (per 2 points increased in score)	General population	8	NR	0.92 (0.90–0.94)	33	No	Critically low
Liyanage[11]	2016	RCT	High risk population	5	10671	1.00 (0.86–1.15)	NR	NR	Critically low
				Mediterranean diet (CVD mortality)					
Sofi[6]	2008	Cohort (per 2 points increased in score)	General population	3	NR	0.91 (0.87–0.95)	32.6	NR	Critically low
Sofi[7]	2010	Cohort (per 2 points increased in score)	General population	6	NR	0.90 (0.87–0.93)	35	No	Critically low

	Year	Types of study	Population	No. of primary studies	Total n	Pooled RR (95% CI)	Heterogeneity, I^2	Publication bias	Risk of bias
Liyanage[11]	2016	RCT	High risk population	4	10623	0.90 (0.72–1.11)	NR	NR	Critically low
Grosso[12]	2017	Cohort (highest vs lowest)	General population	13	NR	0.75 (0.68–0.83)	75%	NR	Critically low
Grosso[12]	2017	RCT	General population	4	NR	0.59 (0.38–0.93)	46	NR	Critically low
Rosato[13]	2019	Cohort (highest vs lowest)	General population	7	NR	0.73 (0.67–0.81)	47.1	NR	Critically low
				Mediterranean diet (CVD)					
Martínez-González[9]	2014	RCT	General population	2	NR	0.64 (0.53–0.79)	54.5	NR	Critically low
Liyanage[11]	2016	RCT	High risk population	3	9052	0.63 (0.53–0.75)	NR	NR	Critically low
Grosso[12]	2017	Cohort (highest vs lowest)	General population	13	NR	0.73 (0.66–0.80)	36%	NR	Critically low
Grosso[12]	2017	RCT	General population	4	NR	0.55 (0.39–0.76)	38	No	Critically low
Rosato[13]	2019	Cohort (highest vs lowest)	General population	11	54894	0.81 (0.74–0.88)	79.9	NR	Critically low
				Mediterranean diet (CHD)					
Liyanage[11]	2016	RCT	High risk population	3	9052	0.65 (0.50–0.85)	NR	NR	Critically low
Grosso[12]	2017	Cohort (highest vs lowest)	General population	4	NR	0.72 (0.60–0.86)	No	NR	Critically low
Rosato[13]	2019	Cohort and case control (highest vs lowest)	General population	11	NR	0.70 (0.62–0.82)	44.5	Yes	Critically low
				Mediterranean diet (Stroke)					
Psaltopoulou[8]	2013	Cohort (highest vs lowest)	General population	12	NR	0.71 (0.57–0.89)	69.1	No	Critically low
Kontogianni[10]	2014	Cohort (highest vs lowest)	General population	NR	195,875	0.68 (0.58–0.79)	0.0	NR	Critically low
Chen[14]	2019	Cohort (every increased 4 points in MedDiet score)	General population	20	NR	0.84 (0.81–0.88)	11.5	Yes	Critically low
Liyanage[11]	2016	RCT	High risk population	3	9052	0.65 (0.48–0.88)	NR	NR	Critically low
Grosso[12]	2017	Cohort (highest vs lowest)	General population	5	NR	0.76 (0.60–0.96)	52%	NR	Critically low
Grosso[12]	2017	RCT	General population	2	NR	0.64 (0.47–0.86)	0	NR	Critically low

	Year	Types of study	Population	No. of primary studies	Total n	Pooled RR (95% CI)	Heterogeneity, I^2	Publication bias	Risk of bias
Rosato[13]	2019	Cohort and case control (highest vs lowest)	General population	5	2997	0.82 (0.73–0.92)	0	yes	Critically low
DASH diet (CHD)									
Abargouei[15]	2013	Cohort (highest vs lowest)	General population	3	144,337	0.79 (0.71–0.88)	0	Yes	Critically low
Yang[18]	2019	Cohort (highest vs lowest)	General population	7	NR	0.82 (0.78–0.87)	0	No	Critically low
Yang[18]	2019	Every 4 points increased in DASH diet score	General population	7	NR	0.95 (0.94–0.97)	NR	No	Critically low
DASH diet (Stroke)									
Abargouei[15]	2013	Cohort (highest vs lowest)	General population	3	150,191	0.81 (0.72–0.92)	0	Yes	Critically low
Feng[17]	2018	Cohort (highest vs lowest)	General population	11	474228	0.88 (0.83–0.93)	4	Yes	Critically low
Vegetarian diet (CVD mortality)									
Kwok[16]	2014	Cohort (highest vs lowest)	General population	7	NR	0.87 (0.68–1.11)	97	NR	Critically low
Vegetarian diet (CHD)									
Kwok[16]	2014	Cohort (highest vs lowest)	General population	7	NR	0.71 (0.57–0.87)	83	NR	Critically low
Vegetarian diet (Stroke)									
Kwok[16]	2014	Cohort (highest vs lowest)	General population	6	NR	0.93 (0.70–1.23)	79	NR	Critically low
Vegetables and fruits (CVD mortality)									
Kim[48]	2016	Vegetables; Cohort (highest vs lowest)	General population	3	NR	0.95 (0.89–1.02)	NR	NR	Critically low
Kim[48]	2016	Fruits; Cohort (highest vs lowest)	General population	3	NR	0.96 (0.83–1.11)	NR	NR	Critically low
Vegetables and fruits (CHD)									
Law[19]	1998	Fruits; Cohort (highest vs lowest)	General population	3	NR	0.86 (0.71–1.05)	Yes	NR	Critically low
Law[19]	1998	Vegetables; Cohort (highest vs lowest)	General population	2	NR	0.82 (0.66–1.02)	Yes	NR	Critically low
Vegetables and fruits (Stroke)									
Hu[20]	2014	Fruits; Cohort (highest vs lowest)	General population	19	NR	0.77 (0.71–0.84)	52.3	Yes	Critically low
Hu[20]	2014	Vegetables; Cohort (highest vs lowest)	General population	16	NR	0.86 (0.79–0.93)	40.3	Yes	Critically low
Nuts (CHD)									

	Year	Types of study	Population	No. of primary studies	Total n	Pooled RR (95% CI)	Heterogeneity, I^2	Publication bias	Risk of bias
Weng[27]	2016	Cohort (per 1 serving/week)	General population	13	NR	0.90 (0.87–0.94)	68.2	NR	Critically low
Weng[27]	2016	Cohort (highest vs lowest)	General population	14	NR	0.68 (0.59–0.78)	62.7	No	Critically low
Nut (stroke)									
Shao[26]	2016	Cohort (highest vs lowest)	General population	11	NR	0.88 (0.80–0.97)	0.0	No	Low
Shao[26]	2016	Cohort (per 12 g/day)	General population		NR	0.86 (0.79–0.94)	NR	NR	Low
Whole grains (all-cause mortality)									
Wei[28]	2016	Cohort (highest vs lowest)	General population	11	NR	0.87 (0.84–0.90)	67.3	Yes	Critically low
Wei[28]	2016	Cohort (per 3 servings/day)	General population	10	NR	0.81 (0.76–0.85)	79.3	NR	Critically low
Reynolds[50]	2019	Cohort (highest vs lowest)	General population	9	10·7 million person-years	0·81 (0·72–0·90)	97.4	No	low
Whole grain (CVD mortality)									
Wei[28]	2016	Cohort (highest vs lowest)	General population	9	NR	0.81 (0.74–0.89)	56.9	No	Critically low
Wei[28]	2016	Cohort (per 3 servings/day)	General population	8	NR	0.74 (0.66–0.83)	76.3	NR	Critically low
Reynolds[50]	2019	Cohort (highest vs lowest)	General population	2	2·0 million person-years	0.66 (0·56–0.77)	47.9	No	low
Whole grain (CHD)									
Reynolds[50]	2019	Cohort Highest vs lowest)	General population	6	2·8 million person-years	0.80 (0.70–0.91)	79.1	No	low
Whole grain (Stroke)									
Reynolds[50] 350	2019	Cohort (highest vs lowest)	General population	3	1·1 million person-years	0·86 (0·61 to 1·21)	65.2	No	low
Legumes (CVD mortality)									
Kim[48]	2016	Cohort (highest vs lowest)	General population	2	NR	0.89 (0.82–0.98)	NR	NR	Critically low
Legumes (CHD)									
Marventano[29]	2016	Cohort (highest vs lowest)	General population	12	NR	0.90 (0.84–0.97)	34	No	Critically low
Legumes (stroke)									
Marventano[29]	2016	Cohort (highest vs lowest)	General population	8	NR	1.01 (0.89–1.14)	59	No	Critically low
Fish intake (CVD mortality)									
Whelton[21]	2004	Cohort (per 2–<4 portions/week)	General population	11	NR	0.75 (0.62–0.92)	NR	NR	Critically low

	Year	Types of study	Population	No. of primary studies	Total n	Pooled RR (95% CI)	Heterogeneity, I^2	Publication bias	Risk of bias
Skeaff[32]	2009	Cohort (highest vs lowest)	General population	22	317988	0.82 (0.71, 0.94)	51.6	NR	Critically low
				Fish intake (CHD)					
Whelton[21]	2004	Cohort (per 2–<4 portion/week)	General population	9	NR	0.83 (0.69–0.99)	NR	NR	Critically low
Skeaff[32]	2009	Cohort (highest vs lowest)	General population	7	204734	0.87 (0.71, 1.06)	70.7	NR	Critically low
				Olive oil (CHD)					
Martínez-González[37]	2014	Cohort (highest vs lowest) and RCT	General population	4	NR	0.94 (0.78–1.14)	66	Yes	Critically low
				Olive oil (stroke)					
Martínez-González[37]	2014	Cohort (highest vs lowest) and RCT	General population	3	NR	0.76 (0.67–0.86)	0	No	Critically low
				Chocholate (CVD mortality)					
Kwok[23]	2015	Cohort (highest vs lowest)	General population	3	NR	0.55 (0.36–0.83)	67	NR	Critically low
				Chocholate (CHD)					
Kwok[23]	2015	Cohort (highest vs lowest)	General population	5	NR	0.71 (0.56–0.92)	61	NR	Critically low
Yuan[24]	2017	Cohort (highest vs lowest)	General population	6	NR	0.90 (0.82–0.97)	24.3	No	Critically low
				Chocholate (stroke)					
Kwok[23]	2015	Cohort (highest vs lowest)	General population	5	NR	0.79 (0.70–0.87)	0	NR	Critically low
Yuan[24]	2017	Cohort (highest vs lowest)	General population	8	NR	0.84 (0.78–0.90)	0	Yes	Critically low
				Coffee (all-cause mortality)					
Crippa[22]	2014	Cohort (per 1 cup/day)	General population	15	NR	0.92 (0.91–0.94)	58.1	No	Critically low
				Coffee (CVD mortality)					
Crippa[22]	2014	Cohort (per 1 cup/day)	General population	13	NR	0.89 (0.86–0.91)	58.8	No	Critically low
				Green tea (all-cause mortality)					
Pang[25]	2015	Cohort (1-3 cup vs <1 cups/day)	General population	2	31196	0.79 (0.47–1.32)	91	No	Critically low
				Green tea (CVD mortality)					
Pang[25]	2015	Cohort (1-3 cup vs <1 cups/day)	General population	1	27894	0.88 (0.75–1.03)	NR	No	Critically low
				Green tea (CVD)					

	Year	Types of study	Population	No. of primary studies	Total n	Pooled RR (95% CI)	Heterogeneity, I²	Publication bias	Risk of bias
Pang[25]	2015	Cohort (1-3 cup vs <1 cups/day)	General population	2	41755	0.93 (0.85–1.01)	0	No	Critically low
				Green tea (Stroke)					
Pang[25]	2015	Cohort (1-3 cup vs <1 cups/day)	General population	5	67884	0.64 (0.47–0.86)	87	No	Critically low
				Total fat intake (all-cause mortality)					
Hooper[30]	2000	RCT	General population	NR	NR	0.98 (0.86–1.12)	No	Yes	Critically low
Harcombe[40]	2016	RCT	General population	10	62421	0.99 (0.94–1.05)	NR	NR	Critically low
				Total fat intake (CVD mortality)					
Hooper[30]	2000	RCT	General population	NR	NR	0.91 (0.77–1.07)	No	NR	Critically low
Skeaff[32]	2009	RCT	General population	2	46810	1.00 (0.80, 1.24)	0	NR	Critically low
Skeaff[32]	2009	Cohort (highest vs lowest)	General population	6	45416	0.94 (0.74, 1.18)	62.1	NR	Critically low
Harcombe[40]	2016	RCT	General population	10	62421	0.98 (0.88–1.08)	NR	NR	Critically low
Harcombe[41]	2017	Cohort (highest vs lowest)	General population	6	NR	1.04 (0.98–1.10)	56.09	No	Critically low
				Total fat intake (CHD)					
Skeaff[32]	2009	RCT	General population	2	46810	0.93 (0.84, 1.04)	0	NR	Critically low
Skeaff[32]	2009	Cohort (highest vs lowest)	General population	4	147403	0.93 (0.84, 1.03)	0	NR	Critically low
				Saturated Fat (CVD mortality)					
Skeaff[32]	2009	Cohort (highest vs lowest)	General population	8	80655	1.14 (0.82, 1.60)	72.1	NR	Critically low
Harcombe[41]	2017	Cohort (highest vs lowest)	General population	6	NR	1.08 (0.94–1.25)	78.43	No	Critically low
				Saturated Fat (CHD)					
Skeaff[32]	2009	Cohort (highest vs lowest)	General population	5	147818	0.93 (0.83, 1.05)	0.09	NR	Critically low
				Saturated Fat (Stroke)					
Muto[42]	2018	Cohort (highest vs lowest)	General population	11	NR	0.89 (0.82, 0.96)	38.9	Yes	Critically low
				Trans fatty acid (CVD mortality)					
Skeaff[32]	2009	Cohort (highest vs lowest)	General population	3	68625	1.32 (1.08, 1.61)	0	NR	Critically low

	Year	Types of study	Population	No. of primary studies	Total n	Pooled RR (95% CI)	Heterogeneity, I^2	Publication bias	Risk of bias
				Trans fatty acid (CHD)					
Skeaff[32]	2009	Cohort (highest vs lowest)	General population	5	148070	1.25 (1.07, 1.46)	44	NR	Critically low
				MUFA (all-cause mortality)					
Schwingshackl[38]	2014	Cohort (highest vs lowest)	General population	17	NR	0.89 (0.83–0.96)	64	Yes	Critically low
				MUFA (CVD mortality)					
Skeaff[32]	2009	Cohort (highest vs lowest)	General population	5	25681	0.85 (0.60, 1.20)	70.9	NR	Critically low
Schwingshackl[38]	2014	Cohort (highest vs lowest)	General population	14	NR	0.88 (0.80–0.96)	50	Yes	Critically low
				MUFA (CHD)					
Skeaff[32]	2009	Cohort (highest vs lowest)	General population	3	103646	0.87 (0.74, 1.03)	27.4	NR	Critically low
Schwingshackl[38]	2014	Cohort (highest vs lowest)	General population	15	NR	0.96 (0.90–1.01)	41	No	Critically low
				MUFA (Stroke)					
Schwingshackl[38]	2014	Cohort (highest vs lowest)	General population	11	NR	0.83 (0.71–0.97)	70	No	Critically low
				n-3 PUFA (All-cause mortality)					
Lista[35]	2012	RCT	General population	17	50468	0.95 (0.89–1.02)*	28	NR	Critically low
Abdelhamid[36]	2019	RCT	General population	39	92653	0.98 (0.93–1.03)	11.64	Yes	High
				n-3 PUFA (CVD mortality)					
Skeaff[32]	2009	RCT	General population	12	37369	0.88 (0.76–1.01)	11.5	NR	Critically low
Lista[35]	2012	RCT	General population	13	46737	0.91 (0.83–0.99)*	32	NR	Critically low
Abdelhamid[36]	2019	RCT	General population	25	67772	0.95 (0.87–1.03)	24	Yes	High
				n-3 PUFA (CVD)					
Skeaff[32]	2009	RCT	General population	7	14659	0.89 (0.82–0.98)	0	NR	Critically low
Lista[35]	2012	RCT	General population	14	45285	0.90 (0.85–0.96)	53	NR	Critically low
Abdelhamid[36]	2019	RCT	General population	38	90378	0.99 (0.94–1.04)	37	Yes	High
n-3 PUFA (CHD)									
Abdelhamid[36]	2019	RCT	General population	28	84301	0.93 (0.88–0.97)	0	Yes	High
n-3 PUFA (Stroke)									
Abdelhamid[36]	2019	RCT	General population	28	89358	1.06 (0.97–1.16)	0	No	High
				n-6 PUFA (all-cause mortality)					

	Year	Types of study	Population	No. of primary studies	Total n	Pooled RR (95% CI)	Heterogeneity, I^2	Publication bias	Risk of bias
Ramsden[34]	2010	RCT	General population	4	NR	1.16 (0.95–1.42)	NR	NR	Critically low
				n-6 PUFA (CVD mortality)					
Mozaffarian[33]	2010	RCT	General population	8	13614	0.81 (0.70, 0.95)	37	Yes	Low
Ramsden[34]	2010	RCT	General population	3	NR	1.17 (0.82–1.68)	NR	NR	Critically low
				n-6 PUFA (CHD)					
Skeaff[32]	2009	Cohort (highest vs lowest)	General population	3	66102	1.05 (0.92, 1.20)	0	NR	Critically low
Farvid[39]	2015	Cohort (highest vs lowest)	General population	14	NR	0.85 (0.78–0.92)	35.5	No	Critically low
				Combined n-3, n-6 PUFA (all-cause mortality)					
Ramsden[34]	2010	RCT	General population	4	NR	0.92 (0.80–1.06)	NR	NR	Critically low
				Combined n-3, n-6 PUFA (CVD mortality)					
Skeaff[32]	2009	RCT	General population	6	4528	0.84 (0.62, 1.12)	12.4	NR	Critically low
Ramsden[34]	2010	RCT	General population	4	NR	0.81 (0.64–1.03)	NR	NR	Critically low
				Combined n-3, n-6 PUFA (CHD)					
Skeaff[32]	2009	RCT	General population	9	15085	0.83 (0.69, 1.00)	44.2	NR	Critically low
				Fibre intake (All-cause mortality)					
Reynolds[50] 350	2019	Cohort (highest vs lowest)	General population	10	12.3 millions person-year	0.85 (0.79–0.91)	75.8	NR	low
				Fibre intake (CVD mortality)					
Kim[48]	2016	Cohort (highest vs lowest)	General population	8	NR	0.76 (0.67–0.87)	40.1	No	Critically low
Kim[48]	2016	Cohort (per 10 g/day)	General population	8	NR	0.91 (0.88–0.94)	30.8	No	Critically low
Reynolds[50] 350	2019	Cohort (highest vs lowest)	General population	10	6·9 million person-years	0·69 (0·60 to 0·81)	51.3	Yes	low
				Fibre intake (CHD)					
Law[19]	1998	Fruit fiber; cohort (highest vs lowest)	General population	3	NR	0.86 (0.79–0.95)	Yes	NR	Critically low
Law[19]	1998	Vegetable fiber; cohort (highest vs lowest)	General population	3	NR	0.81 (0.71–0.93)	Yes	NR	Critically low
Reynolds[50] 350	2019	Cohort (highest vs lowest)	General population	9	2·7 million person-years	0·76 (0·69 to 0·83)	14.3	No	low
				Fibre intake (Stroke)					

	Year	Types of study	Population	No. of primary studies	Total n	Pooled RR (95% CI)	Heterogeneity, I^2	Publication bias	Risk of bias
Chen[47]	2013	Cohort (highest vs lowest)	Stroke	6	279253	0.87 (0.77–0.99)	36.4	No	Critically low
Chen[47]	2013	Cohort (per 10 g/day)	Stroke	NR	NR	0.88 (0.79–0.97)	47.6	NR	Critically low
Li[49]	2017	Cohort (highest vs lowest)	Ischemic stroke	8	NR	0.85 (0.79–0.91)	4.5	No	Critically low
Reynolds[50]	2019	Cohort (highest vs lowest)	General population	9	4·6 million person-years	0·78 (0·69–0·88)	38.5	No	low
Low salt diet (all-cause mortality)									
Aburto[51]	2013	Cohort and RCT (lowest vs highest)	General population	7	21515	0.94 (0.83–1.06)	61	NR	Critically low
Adler[52]	2014	RCT	Hypertensive patients	7	6603	0.96 (0.83–1.10)	0.0	NR	Low
Low salt diet (CVD mortality)									
Aburto[51]	2013	Cohort and RCT (lowest vs highest)	General population	7	41881	0.93(0.75–1.15)	80	NR	Critically low
Adler[52]	2014	RCT	Hypertensive patients	3	2656	0.67 (0.45–1.01)	0.0	NR	Low
Low salt diet (CVD)									
Aburto[51]	2013	Cohort and RCT (lowest vs highest)	General population	9	46483	0.89 (0.75–1.08)	78	NR	Critically low
Adler[52]	2014	RCT	Hypertensive patients	4	3397	0.76 (0.57–1.01)	0.0	NR	Low
Low salt diet (stroke)									
Aburto[51]	2013	Cohort and RCT (lowest vs highest)	General population	10	72878	0.81 (0.70–0.93)	49	NR	Critically low
Jayedi[53]	2019	Cohort (every 1g/d decreased in sodium intake)	General population	14	253449	0.94 (0.90–0.98)	60.4	Yes	Critically low
Flavonoid intake (all-cause mortality)									
Kim[44]	2017	Cohort (highest vs lowest)	General population	8	NR	0.86 (0.73–1.00)	67.6	No	Critically low
Flavonoid intake (CVD mortality)									
Kim[44]	2017	Cohort (highest vs lowest)	General population	13	NR	0.86 (0.75–0.98)	50.6	No	Critically low
Kimble[45]	2019	Cohort (highest vs lowest)	General population	7	NR	0.92 (0.87–0.97)	0	No	Critically low
Flavonoid intake (CHD)									
Kimble[45]	2019	Cohort (highest vs lowest)	General population	5	NR	0.91 (0.83–0.99)	12.0	No	Critically low
Flavonoid intake (stroke)									
Hollman[43]	2010	Cohort (highest vs lowest)	General population	6	NR	0.80 (0.65–0.98)	54	NR	Critically low

	Year	Types of study	Population	No. of primary studies	Total n	Pooled RR (95% CI)	Heterogeneity, I^2	Publication bias	Risk of bias
Kimble[45]	2019	Cohort (highest vs lowest)	General population	10	NR	1.00 (0.93–1.07)	15.1	No	Critically low
Potassium (CHD)									
D'Elia[46]	2011	Cohort (highest vs lowest)	General population	6	81612	0.92 (0.81–1.04)	45	No	Critically low
Potassium (stroke)									
D'Elia[46]	2011	Cohort (highest vs lowest)	General population	11	233606	0.79 (0.68–0.90)	55	No	Critically low
Calcium intake (all-cause mortality)									
Wang[54]	2014	Cohort (highest vs lowest)	General population	6	225189	0.83 (0.70–1.00)	74.9	No	Critically low
Calcium intake (CVD mortality)									
Wang[54]	2014	Cohort (highest vs lowest)	General population	9	709499	0.97 (0.89–1.07)	18.8	No	Critically low

*Pooled OR; AHEI: alternate healthy eating index; CVD: cardiovascular diseases; CHD: coronary heart disease; CVH: cardiovascular health; DII: dietary inflammatory index; DM: diabetes mellitus; DASH: Dietary Approaches to Stop Hypertension; HT: hypertension; HEI: healthy eating index; NR: not reported; MUFA: monounsaturated fatty acid; PUFA: polyunsaturated fatty acid; RCT: randomized-controlled trial; SFA: saturated fatty acid.

Table S4. Results of methodological quality assessment, according to AMSTAR 2.

Author, year	1	2	3	4	5	6	7	8	9	10	11	12	13	14	15	16	Confidence
Onvani[1], 2016	Yes	No	No	Partial yes	Yes	Yes	No	Partial yes	Yes	No	Yes	No	No	Yes	Yes	No	Critically low
Semlitsch[2], 2016	Yes	No	Yes	Yes	Yes	Yes	Yes	Yes	Yes	Yes	Yes	Yes	Yes	Yes	Yes	Yes	Low
Guo[3], 2017	Yes	No	No	Partial yes	No	Yes	No	Partial yes	Yes	No	Yes	Yes	Yes	Yes	Yes	Yes	Critically low
Schwingshackl[4], 2018	No	Yes	Yes	Partial yes	No	No	No	Partial yes	Yes	No	Yes	No	No	Yes	Yes	Yes	Critically low
Shivappa[5], 2018	Yes	No	No	Partial yes	Yes	No	No	Partial yes	Yes	No	Yes	No	No	Yes	Yes	Yes	Critically low
Sofi[6], 2008	Yes	No	No	Partial yes	No	Yes	No	Partial yes	Partial yes	No	Yes	Yes	Yes	Yes	Yes	Yes	Critically low
Sofi[7], 2010	Yes	No	No	Partial yes	Yes	Yes	No	Partial yes	Partial yes	No	Yes	No	No	Yes	Yes	Yes	Critically low
Psaltopoulou[8], 2013	Yes	No	No	Partial yes	No	Yes	No	Partial yes	Yes	No	Yes	No	No	Yes	Yes	Yes	Critically low
Martínez-González[9], 2014	No	No	Yes	Partial yes	No	No	No	Partial yes	No	No	No	No	No	Yes	Yes	Yes	Critically low
Kontogianni[10], 2014	Yes	No	Yes	Partial yes	No	No	No	No	No	No	Yes	No	No	Yes	No	Yes	Critically low
Liyanage[11], 2016	Yes	Partial yes	Yes	Partial yes	Yes	Yes	Partial yes	Yes	Yes	No	Yes	Yes	Yes	No	No	Yes	Critically low
Grosso[12], 2017	Yes	No	No	Partial yes	Yes	No	No	Yes	Yes	No	Yes	Yes	Yes	Yes	Yes	Yes	Critically low
Abargouei[15], 2013	Yes	No	Yes	Partial yes	Yes	Yes	No	Partial yes	No	No	Yes	No	No	Yes	Yes	Yes	Critically low
Kwok[16], 2014	Yes	No	No	Partial yes	Yes	Yes	No	Yes	Partial yes	No	Yes	No	Yes	Yes	No	Yes	Critically low
Law[19], 1998	Yes	No	No	Partial yes	No	No	No	Partial yes	No	No	Yes	No	No	Yes	No	No	Critically low
Hu[20], 2014	Yes	No	Yes	Partial yes	No	No	No	Partial yes	Yes	No	Yes	Yes	Yes	Yes	Yes	Yes	Critically low
Whelton[21], 2004	Yes	No	Yes	Partial yes	No	No	No	Partial yes	No	No	Yes	No	No	No	No	No	Critically low
Crippa[22], 2014	Yes	No	No	Partial yes	Yes	No	Yes	Partial yes	No	No	Yes	No	No	Yes	Yes	Yes	Critically low
Kwok[23], 2015	Yes	No	Yes	Partial yes	Yes	Yes	No	Partial yes	Yes	No	Yes	Yes	Yes	Yes	No	Yes	Critically low

Yuan[24], 2017	Yes	No	No	Partial yes	Yes	Yes	No	Partial yes	Yes	No	Yes	Yes	Yes	Yes	Yes	Yes	Critically low
Pang[25], 2015	Yes	No	No	Partial yes	Yes	Yes	No	Yes	No	No	Yes	No	No	Yes	Yes	Yes	Critically low
Shao[26], 2016	Yes	No	No	Partial yes	No	Yes	Yes	Yes	Yes	No	Yes	Yes	Yes	Yes	Yes	Yes	Low
Weng[27], 2016	Yes	No	No	Partial yes	No	Yes	No	Partial yes	No	No	Yes	No	No	Yes	Yes	Yes	Critically low
Wei[28], 2016	Yes	No	No	Partial yes	Yes	Yes	No	Yes	Yes	No	Yes	Yes	Yes	Yes	Yes	Yes	Critically low
Marventano[29], 2016	Yes	No	No	Partial yes	Yes	No	No	No	Yes	No	Yes	Yes	Yes	Yes	Yes	Yes	Critically low
Hooper[30], 2000	Yes	No	No	Yes	Yes	Yes	Partial yes	Yes	No	No	Yes	No	No	Yes	Yes	No	Critically low
Studer[31], 2005	Yes	No	Yes	No	Yes	Yes	Partial yes	Partial yes	Partial yes	No	Yes	Yes	Yes	Yes	No	Yes	Critically low
Skeaff[32], 2009	Yes	No	No	Partial yes	No	No	No	Partial yes	No	No	Yes	No	No	Yes	Yes	No	Critically low
Mozaffarian[33], 2010	Yes	Partial yes	Yes	Yes	Yes	Yes	Yes	Partial yes	Partial yes	No	Yes	Yes	Yes	Yes	Yes	Yes	Low
Ramsden[34], 2010	Yes	No	Yes	Partial yes	No	No	No	Yes	No	No	Yes	No	No	No	Yes	Yes	Critically low
Lista[35], 2012	Yes	No	Yes	Partial yes	Yes	No	No	Partial yes	Partial yes	No	Yes	Yes	Yes	Yes	No	No	Critically low
Martínez-González[37], 2014	Yes	No	No	Partial yes	No	Yes	No	Partial yes	Yes	No	Yes	No	No	Yes	Yes	Yes	Critically low
Schwingshackl[38], 2014	Yes	No	No	Partial yes	Yes	No	No	Partial yes	Yes	No	Yes	No	No	Yes	Yes	Yes	Critically low
Farvid[39], 2015	Yes	No	No	Yes	Yes	Yes	No	Partial yes	No	No	Yes	No	No	Yes	Yes	Yes	Critically low
Harcombe[40], 2016	Yes	No	Yes	No	Yes	No	No	Partial yes	Yes	No	Yes	No	Yes	Yes	Yes	Yes	Critically low
Harcombe[41], 2017	Yes	No	Yes	Partial yes	Yes	No	No	Partial yes	Yes	No	Yes	Yes	No	Yes	Yes	No	Critically low
Muto[42], 2018	Yes	No	No	No	Yes	No	No	Partial yes	No	No	Yes	No	No	Yes	Yes	No	Critically low
Hollman[43], 2010	Yes	No	Yes	Partial yes	No	Yes	No	Yes	No	No	Yes	No	No	Yes	Yes	Yes	Critically low
Kim[44], 2017	Yes	No	No	Partial yes	No	Yes	No	Partial yes	Yes	No	Yes	Yes	Yes	Yes	Yes	Yes	Critically low
D'Elia[46], 2011	Yes	No	Yes	Partial yes	Yes	Yes	No	Partial yes	Yes	No	Yes	Yes	Yes	Yes	Yes	Yes	Critically low

Chen[47], 2013	Yes	No	No	Partial yes	Yes	Yes	No	Yes	Yes	No	Yes	Yes	Yes	Yes	Yes	Yes	Critically low
Kim[48], 2016	Yes	No	Yes	Partial yes	No	Yes	No	Partial yes	Yes	No	Yes	Yes	Yes	Yes	Yes	Yes	Critically low
Li[49], 2017	Yes	No	Yes	Partial yes	Yes	Yes	No	Partial yes	Yes	No	No	No	No	Yes	Yes	Yes	Critically low
Aburto[51], 2013	Yes	No	Yes	Yes	Yes	Yes	No	No	Yes	No	Yes	Yes	Yes	Yes	Yes	Yes	Critically low
Adler[52], 2014	Yes	Partial yes	Yes	Yes	Yes	Yes	Yes	Yes	Yes	Yes	Yes	Yes	Yes	Yes	No	No	Low
Wang[54], 2014	Yes	No	No	Partial yes	Yes	Yes	No	Partial yes	Yes	No	Yes	Yes	Yes	Yes	Yes	Yes	Critically low
Abdelhamid[36]	Yes	Yes	Yes	Yes	Yes	Yes	Yes	Yes	Yes	Yes	Yes	Yes	Yes	Yes	Yes	Yes	High
Kimble[45]	Yes	Yes	Yes	Partial yes	No	Yes	Partial yes	Partial yes	Yes	Yes	No	No	No	Yes	Yes	No	Critically low
Yang[18]	Yes	No	No	Partial yes	No	Yes	Partial yes	Yes	Yes	No	Yes	No	No	Yes	Yes	Yes	Critically low
Jayedi[53]	Yes	No	Yes	Partial yes	Yes	Yes	Partial yes	Yes	Yes	Yes	Yes	No	No	Yes	Yes	Yes	Critically low
Chen[14]	Yes	No	No	Yes	Yes	No	Partial yes	Yes	Yes	Yes	Yes	No	No	Yes	Yes	Yes	Critically low
Reynolds[50],2019 No 350	Yes	Partial yes	Yes	Yes	Yes	No	No	No	RCT= yes Observational=No	No	Yes	Yes	Yes	Yes	Yes	Yes	Low
Rosato[13], 2019 No 351	Yes	No	Yes	No	Yes	Yes	Yes	Partial yes	No	No	Yes	No	No	Yes	Yes	Yes	Critically low
Feng[17], 2018 No 491	Yes	No	Yes	Partial Yes	No	Yes	No	Partial yes	No	Yes	Yes	Yes	Yes	Yes	Yes	No	Critically low

Reference

1. Onvani S, Haghighatdoost F, Surkan PJ, et al. Adherence to the Healthy Eating Index and Alternative Healthy Eating Index dietary patterns and mortality from all causes, cardiovascular disease and cancer: a meta-analysis of observational studies. *J Hum Nutr Diet* 2017;30(2):216-26. doi: 10.1111/jhn.12415
2. Semlitsch T, Jeitler K, Berghold A, et al. Long-term effects of weight-reducing diets in people with hypertension. *Cochrane Database Syst Rev* 2016;3:Cd008274. doi: 10.1002/14651858.CD008274.pub3
3. Guo L, Zhang S. Association between ideal cardiovascular health metrics and risk of cardiovascular events or mortality: A meta-analysis of prospective studies. *Clin Cardiol* 2017;40(12):1339-46. doi: 10.1002/clc.22836
4. Schwingshackl L, Bogensberger B, Hoffmann G. Diet Quality as Assessed by the Healthy Eating Index, Alternate Healthy Eating Index, Dietary Approaches to Stop Hypertension Score, and Health Outcomes: An Updated Systematic Review and Meta-Analysis of Cohort Studies. *J Acad Nutr Diet* 2018;118(1):74-100.e11. doi: 10.1016/j.jand.2017.08.024
5. Shivappa N, Godos J, Hébert JR, et al. Dietary inflammatory index and cardiovascular risk and mortality—a meta-analysis. *Nutrients* 2018;10(2) doi: 10.3390/nu10020200
6. Sofi F, Cesari F, Abbate R, et al. Adherence to Mediterranean diet and health status: meta-analysis. *Bmj* 2008;337:a1344. doi: 10.1136/bmj.a1344
7. Sofi F, Abbate R, Gensini GF, et al. Accruing evidence on benefits of adherence to the Mediterranean diet on health: an updated systematic review and meta-analysis. *Am J Clin Nutr* 2010;92(5):1189-96. doi: 10.3945/ajcn.2010.29673
8. Psaltopoulou T, Sergentanis TN, Panagiotakos DB, et al. Mediterranean diet, stroke, cognitive impairment, and depression: A meta-analysis. *Ann Neurol* 2013;74(4):580-91. doi: 10.1002/ana.23944
9. Martinez-Gonzalez MA, Bes-Rastrollo M. Dietary patterns, Mediterranean diet, and cardiovascular disease. *Curr Opin Lipidol* 2014;25(1):20-26. doi: 10.1097/MOL.0000000000000044
10. Kontogianni MD, Panagiotakos DB. Dietary patterns and stroke: a systematic review and re-meta-analysis. *Maturitas* 2014;79(1):41-7. doi: 10.1016/j.maturitas.2014.06.014
11. Liyanage T, Ninomiya T, Wang A, et al. Effects of the Mediterranean Diet on Cardiovascular Outcomes-A Systematic Review and Meta-Analysis. *PLoS One* 2016;11(8):e0159252. doi: 10.1371/journal.pone.0159252
12. Grosso G, Marventano S, Yang J, et al. A comprehensive meta-analysis on evidence of Mediterranean diet and cardiovascular disease: Are individual components equal? *Crit Rev Food Sci Nutr* 2017;57(15):3218-32. doi: 10.1080/10408398.2015.1107021
13. Rosato V, Temple NJ, La Vecchia C, et al. Mediterranean diet and cardiovascular disease: a systematic review and meta-analysis of observational studies. *Eur J Nutr* 2019;58(1):173-91. doi: 10.1007/s00394-017-1582-0
14. Chen GC, Neelakantan N, Martín-Calvo N, et al. Adherence to the Mediterranean diet and risk of stroke and stroke subtypes. *Eur J Epidemiol* 2019;34(4):337-49. doi: 10.1007/s10654-019-00504-7
15. Salehi-Abargouei A, Maghsoudi Z, Shirani F, et al. Effects of Dietary Approaches to Stop Hypertension (DASH)-style diet on fatal or nonfatal cardiovascular diseases-Incidence: A systematic review and meta-analysis on observational prospective studies. *Nutrition* 2013;29(4):611-18. doi: 10.1016/j.nut.2012.12.018
16. Kwok CS, Umar S, Myint PK, et al. Vegetarian diet, Seventh Day Adventists and risk of cardiovascular mortality: a systematic review and meta-analysis. *Int J Cardiol* 2014;176(3):680-6. doi: 10.1016/j.ijcard.2014.07.080
17. Feng Q, Fan S, Wu Y, et al. Adherence to the dietary approaches to stop hypertension diet and risk of stroke: A meta-analysis of prospective studies. *Medicine* 2018;97(38) doi: 10.1097/MD.0000000000012450

18. Yang ZQ, Yang Z, Duan ML. Dietary approach to stop hypertension diet and risk of coronary artery disease: a meta-analysis of prospective cohort studies. *Int J Food Sci Nutr* 2019;70(6):668-74. doi: 10.1080/09637486.2019.1570490
19. Law MR, Morris JK. By how much does fruit and vegetable consumption reduce the risk of ischaemic heart disease? *Eur J Clin Nutr* 1998;52(8):549-56.
20. Hu D, Huang J, Wang Y, et al. Fruits and vegetables consumption and risk of stroke: A meta-analysis of prospective cohort studies. *Stroke* 2014;45(6):1613-19. doi: 10.1161/STROKEAHA.114.004836
21. Whelton SP, He J, Whelton PK, et al. Meta-analysis of observational studies on fish intake and coronary heart disease. *Am J Cardiol* 2004;93(9):1119-23. doi: 10.1016/j.amjcard.2004.01.038
22. Crippa A, Discacciati A, Larsson SC, et al. Coffee consumption and mortality from all causes, cardiovascular disease, and cancer: a dose-response meta-analysis. *Am J Epidemiol* 2014;180(8):763-75. doi: 10.1093/aje/kwu194
23. Kwok CS, Boekholdt SM, Lentjes MA, et al. Habitual chocolate consumption and risk of cardiovascular disease among healthy men and women. *Heart* 2015;101(16):1279-87. doi: 10.1136/heartjnl-2014-307050
24. Yuan S, Li X, Jin Y, et al. Chocolate consumption and risk of coronary heart disease, stroke, and diabetes: A meta-analysis of prospective studies. *Nutrients* 2017;9(7) doi: 10.3390/nu9070688
25. Pang J, Zhang Z, Zheng TZ, et al. Green tea consumption and risk of cardiovascular and ischemic related diseases: A meta-analysis. *Int J Cardiol* 2016;202:967-74. doi: 10.1016/j.ijcard.2014.12.176
26. Shao C, Tang H, Zhao W, et al. Nut intake and stroke risk: A dose-response meta-analysis of prospective cohort studies. *Sci Rep* 2016;6:30394. doi: 10.1038/srep30394
27. Weng YQ, Yao J, Guo ML, et al. Association between nut consumption and coronary heart disease: a meta-analysis. *Coron Artery Dis* 2016;27(3):227-32. doi: 10.1097/mca.0000000000000331
28. Wei H, Gao Z, Liang R, et al. Whole-grain consumption and the risk of all-cause, CVD and cancer mortality: a meta-analysis of prospective cohort studies. *Br J Nutr* 2016;116(3):514-25. doi: 10.1017/s0007114516001975
29. Marventano S, Izquierdo Pulido M, Sanchez-Gonzalez C, et al. Legume consumption and CVD risk: a systematic review and meta-analysis. *Public Health Nutr* 2017;20(2):245-54. doi: 10.1017/s1368980016002299
30. Hooper L, Summerbell CD, Higgins JP, et al. Reduced or modified dietary fat for preventing cardiovascular disease. *Cochrane Database Syst Rev* 2001(3):Cd002137. doi: 10.1002/14651858.cd002137
31. Studer M, Briel M, Leimenstoll B, et al. Effect of different antilipidemic agents and diets on mortality: a systematic review. *Arch Intern Med* 2005;165(7):725-30. doi: 10.1001/archinte.165.7.725
32. Skeaff CM, Miller J. Dietary fat and coronary heart disease: summary of evidence from prospective cohort and randomised controlled trials. *Ann Nutr Metab* 2009;55(1-3):173-201. doi: 10.1159/000229002
33. Mozaffarian D, Micha R, Wallace S. Effects on coronary heart disease of increasing polyunsaturated fat in place of saturated fat: A systematic review and meta-analysis of randomized controlled trials. *PLoS Med* 2010;7(3) doi: 10.1371/journal.pmed.1000252
34. Ramsden CE, Hibbeln JR, Majchrzak SF, et al. N-6 Fatty acid-specific and mixed polyunsaturate dietary interventions have different effects on CHD risk: A meta-analysis of randomised controlled trials. *British Journal of Nutrition* 2010;104(11):1586-600. doi: 10.1017/S0007114510004010
35. Delgado-Lista J, Perez-Martinez P, Lopez-Miranda J, et al. Long chain omega-3 fatty acids and cardiovascular disease: A systematic review. *British Journal of Nutrition* 2012;107(SUPPL. 2):S201-S13. doi: 10.1017/S0007114512001596
36. Abdelhamid AS, Brown TJ, Brainard JS, et al. Omega-3 fatty acids for the primary and secondary prevention of cardiovascular disease. *Cochrane Database Syst Rev* 2020;3:Cd003177. doi: 10.1002/14651858.CD003177.pub5 [published Online First: 2020/03/03]

37. Martinez-Gonzalez MA, Dominguez LJ, Delgado-Rodriguez M. Olive oil consumption and risk of CHD and/or stroke: a meta-analysis of case-control, cohort and intervention studies. *Br J Nutr* 2014;112(2):248-59. doi: 10.1017/s0007114514000713
38. Schwingshackl L, Hoffmann G. Monounsaturated fatty acids, olive oil and health status: a systematic review and meta-analysis of cohort studies. *Lipids Health Dis* 2014;13:154. doi: 10.1186/1476-511x-13-154
39. Farvid MS, Ding M, Pan A, et al. Dietary linoleic acid and risk of coronary heart disease: A systematic review and meta-analysis of prospective cohort studies. *Circulation* 2014;130(18):1568-78. doi: 10.1161/CIRCULATIONAHA.114.010236
40. Harcombe Z, Baker JS, DiNicolantonio JJ, et al. Evidence from randomised controlled trials does not support current dietary fat guidelines: a systematic review and meta-analysis. *Open Heart* 2016;3(2):e000409. doi: 10.1136/openhrt-2016-000409
41. Harcombe Z, Baker JS, Davies B. Evidence from prospective cohort studies does not support current dietary fat guidelines: a systematic review and meta-analysis. *Br J Sports Med* 2017;51(24):1743-49. doi: 10.1136/bjsports-2016-096550
42. Muto M, Ezaki O. High dietary saturated fat is associated with a low risk of intracerebral hemorrhage and ischemic stroke in japanese but not in non-Japanese: A review and meta-analysis of prospective cohort studies. *J Atheroscler Thromb* 2018;25(5):375-92. doi: 10.5551/jat.41632
43. Hollman PC, Geelen A, Kromhout D. Dietary flavonol intake may lower stroke risk in men and women. *J Nutr* 2010;140(3):600-4. doi: 10.3945/jn.109.116632
44. Kim Y, Je Y. Flavonoid intake and mortality from cardiovascular disease and all causes: A meta-analysis of prospective cohort studies. *Clinical Nutrition ESPEN* 2017;20:68-77. doi: 10.1016/j.clnesp.2017.03.004
45. Kimble R, Keane KM, Lodge JK, et al. Dietary intake of anthocyanins and risk of cardiovascular disease: A systematic review and meta-analysis of prospective cohort studies. *Crit Rev Food Sci Nutr* 2019;59(18):3032-43. doi: 10.1080/10408398.2018.1509835 [published Online First: 2018/10/03]
46. D'Elia L, Barba G, Cappuccio FP, et al. Potassium intake, stroke, and cardiovascular disease: A meta-analysis of prospective studies. *J Am Coll Cardiol* 2011;57(10):1210-19. doi: 10.1016/j.jacc.2010.09.070
47. Chen GC, Lv DB, Pang Z, et al. Dietary fiber intake and stroke risk: A meta-analysis of prospective cohort studies. *Eur J Clin Nutr* 2013;67(1):96-100. doi: 10.1038/ejcn.2012.158
48. Kim Y, Je Y. Dietary fibre intake and mortality from cardiovascular disease and all cancers: A meta-analysis of prospective cohort studies. *Arch Cardiovasc Dis* 2016;109(1):39-54. doi: 10.1016/j.acvd.2015.09.005
49. Li M, Cui F, Yang F, et al. Association between fiber intake and ischemic stroke risk: A meta-analysis of prospective studies. *International Journal of Clinical and Experimental Medicine* 2017;10(3):4659-68.
50. Reynolds A, Mann J, Cummings J, et al. Carbohydrate quality and human health: a series of systematic reviews and meta-analyses. *Lancet* 2019;393(10170):434-45. doi: 10.1016/S0140-6736(18)31809-9
51. Aburto NJ, Ziolkovska A, Hooper L, et al. Effect of lower sodium intake on health: systematic review and meta-analyses. *Bmj* 2013;346:f1326. doi: 10.1136/bmj.f1326
52. Adler AJ, Taylor F, Martin N, et al. Reduced dietary salt for the prevention of cardiovascular disease. *Cochrane Database Syst Rev* 2014(12):Cd009217. doi: 10.1002/14651858.CD009217.pub3
53. Jayedi A, Ghomashi F, Zargar MS, et al. Dietary sodium, sodium-to-potassium ratio, and risk of stroke: A systematic review and nonlinear dose-response meta-analysis. *Clin Nutr* 2019;38(3):1092-100. doi: 10.1016/j.clnu.2018.05.017
54. Wang X, Chen H, Ouyang Y, et al. Dietary calcium intake and mortality risk from cardiovascular disease and all causes: A meta-analysis of prospective cohort studies. *BMC Med* 2014;12(1):1-10. doi: 10.1186/s12916-014-0158-6
